# Thermoplastic starch (TPS)-based composite films for wastewater treatment: synthesis and fundamental characterization

**DOI:** 10.1186/s13065-023-00998-z

**Published:** 2023-07-24

**Authors:** Khadiga Mohamed Abas, Amina Abdel Meguid Attia

**Affiliations:** Laboratory of Surface Chemistry and Catalysis, National Research Center, 33 El-Bohouth St., Giza, 12622 Egypt

**Keywords:** Thermoplastic starch, Starch citrate, Cellulose nanocrystals, Crystal violet, Adsorption

## Abstract

Modification of starch is a potential basic research aiming to improve its water barrier properties. The general purpose of this study is to manufacture cross-linked iodinated starch citrate (ISC) with a degree of substitution (DS) ≈ 0.1 by modifying native corn starch with citric acid in the presence of iodine as an oxidizing agent. Thermoplastic starch (TPS) was generated with urea as a plasticizer and blended with various concentrations of ISC of (2, 4, 6%) (wt/wt) to obtain (UTPS/ISC_2_, UTPS/ISC_4_, and UTPS/ISC_6_). Nanocomposite film was formed from UTPS/ISC_2_ in presence of stabilized iodinated cellulose nanocrystals UTPS/ISC_2_/SICNCs via gelatinization at a temperature of 80ºC. Water solubility and water vapor release were studied amongst the water barrier features. The fabricated starch-based composite films were evaluated utilizing Fourier Transform Infrared Spectroscopy (FTIR), Scanning Electronic Microscope analysis (SEM), surface area, and tensile measurements. The adsorption of crystal violet (CV) dye onto produced samples was examined in an aqueous solution. The findings revealed that the UTPS/ISC_2_/ISCNCs has 83% crystal violet elimination effectiveness. Moreover, the adsorption isotherms were assessed and figured out to vary in the order of Langmuir > Temkin > Freundlich > Dubinin-Radushkevich.

## Introduction

In recent years, biodegradable plastic has emerged as one of the most inventive and rapidly growing segments of the plastic markets [1]. The most important variables affecting the development of this sector are environmental pressures. Therefore other sources of raw materials must be found to face the decreasing of oil resources, as well as rapidly fluctuating material costs are often influenced by geopolitical events [2]. Polysaccharides as starch (S) have numerous advantages over simulated polymers in the plastic field comprising low price, non-poisonous effect, biodegradability and attainability [3]. Corn has long been the foremost widely available commercial source of starch [4]. Starch has been classified as thermoplastic starch (TPS) in biopolymer films by using various processing techniques such as casting, injection, or low modeling [5]. The native starch (S) is plasticized under optimal conditions of temperature, shear forces, and plasticizers to produce thermoplastic starch (TPS). The most common plasticizer for plasticizing starch is water, because of the rapid retrogradation, and gelatinization of starch with water. Utilizing water as a plasticizer leads to its poor mechanical characteristics and brittleness. As a consequence, additional plasticizers such as urea and glycerol have been frequently employed to boost the processing qualities and performance of starch-based films due to their low cost and lack of harmful properties. By minimizing intermolecular interactions between starch molecules, the usage of plasticizer improves the film’s flexibility, extensibility and conductivity. By virtue of the chemical structure of urea (two amino groups, one carbonyl group), and a tendency to crystallize, it has been shown to suppress retrogradation. Treatment with citric acid improves the starch thermodynamic stability [6]. It can be used as a suitable crosslinking agent for starch biopolymers because the interactions between the carboxylic structures of citric acid and the hydroxyl structures of starch lead to a more tightly bound starch citrate. In conjunction with the thermodynamic stability and relative hydrophobic performance of produced starch citrate (SC), it is gaining prominence for industrial applications [6]. It is evident that citric acid can enhance the manufacturability of thermally plasticized starch to produce (TPS) through three techniques: the chemical reaction among citric acid and starch, the cleavage of the starch molecular chain by virtue of the acidolysis of citric acid, and these can lower the interaction between the chains of starch molecules. Chemically, the incorporation of citric acid into starch can withstand the retrogradation estremely well.

Researchers have found that iodine is an ideal acylating agent in the acetylation of polysaccharides such as dextran, cellulose, and starch [7]. Iodine operates as a Lewis acid catalyst by driving the carbonyl citric acid group which makes it more reactive during synthesis. Additionally, iodine has the flexibility to frame a complex through amylose and amylopectin, which can help to solubilize starch in acetylation reactions [8]. Moreover, it has been shown to quickly catalyze the ester formation between alkohols and anhydrides, and it is solely reduced stoichiometrically with sodium thiosulfate to form iodide at the end of esterification reaction of starch with citric acid to produce starch citrate [9]. The use of cellulose nancrystals (CNCs) to strengthen starch-based films might be a promising approach to enhance mechanical performance and reduce permeability while maintaining the long-term stability of the ultimate composite structure that may induce a strong interfacial adhesion of the cellulose template [10]. The amorphous domains of cellulose are frequently ruptured during the creation of CNCs by selective hydrolysis employing mechanical shear, chemical processing, and enzymatic hydrolysis [11]. In an effort to replace products manufactured from petrochemicals, a variety of polymeric matrices have tested CNC as a reinforcement material because of its nanometric particles with interesting properties like biocompatibility within natural polymers, biodegradability, non-toxicity and high surface area [12].

Industrial wastes with various pollutants have a significant negative impact on biodiversity, the ecological environment, and the characteristic ocean activities. In the midst of these impurities, synthetic dyes such as crystal violet (CV) dye are widely used for cotton, dyeing the silk, manufacture of paints, and printing inks. Crystal violet is carcinogenic, non-biodegradable, and can persist in various environments [13]. Flotation, filtration, sedimentation, coagulation/flocculation of toxic pollutants, ozonation, precipitation, electrochemical methods, and membrane-based separation are some of the documented treatment techniques for minimizing the consequences of wastewater [14]. The detriments of traditional treatments surpass their treatment response as toxic emissions, heavy metal production, high prices, and energy demands make wastewater treatment approaches complex [15]. Adsorption has received interest because of its straightforward treatment technique, compelling efficiency and similarly low cost amongst different water purification techniques [16]. The advancement of low cost adsorbents for wastewater treatment has been prioritized. Wastewater treatment has historically relied heavily on adsorption onto activated carbon and clay [17]. However these adsorbents are common for adsorption, they can be toxic to many living organisms. As a result, alternatives to active carbon and clay are being researched to assure sustainability and environmentally friendly adsorption technologies [17]. Rice husk nanoparticles, magnetic biochar nanocomposite (MBC) prepared from rice husk, and iron oxide nanoparticles (IONPs) were evaluated for removing CV dye from aqueous solutions [18]. Prepared starch-based films can be used as an alternative for removing CV dye from wastewater.

The purpose of the current study is to investigate the physicochemical characteristics of composite films that have been synthesized based on starch as a biopolymer, urea as a plasticizer, citric acid, iodine, and CNCs for starch modification treatment to ameliorate starch stability in aqueous solutions as a substitute for the treatment of crystal violet dye from wastewater. Batch adsorption experiments were carried out to evaluate the efficacy of the composite films created at various initial dye concentrations.

## Materials and methods

### Materials

Corncob feed stocks were obtained from nearby areas in the maritime sub-tropical district (Nile Delta). Cobs were threshed to remove the straw and chaff, then dried to less than 10% moisture content and sieved to eliminate any foreign residues. In an air machine, the crushed material was sanitized to be used as a source for CNCs manufacturing. Citric acid (97%), urea (99%), ethanol (95%), sodium thiosulfate (99%), and iodine crystals (98%) were obtained from Sigma-Aldrich Company. Crystal violet dye was purchased by the Merck Company. Corn starch (S) is a type of starch derived from corn that can be available commercially.

### Synthesis of starch citrate (SC) under iodine as an oxidizing agent

The solid–solid interaction technique using iodine as an oxidizing agent was mentioned in the starch modification procedure as described by Biswas et al. [19] with a minor change. Corn starch (S) was employed as a continuous polymer matrix for the production of composite materials. Two grammes sample of native corn starch (S) was placed in a porcelain pot and positioned in a sealed glass jar, which included iodine crystals. The pot of iodine was placed in an air oven at 100 °C for 15 min. The starch particles could not be detached before the system was cooled to room temperature [20]. The blue color of the iodinated starch particles has been achieved. Iodinated starch particles mixed with citric acid at a ratio of 1:1 (*wt/wt*) were heated at 100 °C for 15 min in a muffle furnace, cooled, and mixed with 2 mL of saturated sodium thiosulfate solution with stirring till the color of the mixture had been changed to colorless, indicating the alteration of iodine to iodide. The mixture was gushed into 50 mL of ethanol and stirred for 1 h. The produced iodinated starch citrate (ISC) was filtered, rinsed with distilled water and ethanol to uptake non-reactive citric acid, and dried overnight in a vacuum oven at 60 °C.

### Determination of the produced ISC’s degree of substitution (DS)

The persistance of the degree of substitution (DS) of the ISC sample implicated the entire hydrolysis of the ester bond and titration of the surplus alkali, as reported by Wurzburg [21]. Precisely, 0.5 g of native S (blank sample) and ISC were blended to 30 mL of a 75% ethanol solution. Both samples were agitated in a 50 ºC water bath for 30 min. Following the cooling system, the slurry was brought to room temperature before being bosted with 10 mL of 0.5 N NaOH solution and agitated irregularly for 72 h. Extra alkali in the solution was titrated with 0.2 N HCl, employing phenolphthalein (ph.ph), as an indicator. The acetyl content (%Acetyl) was calculated as follows:

1 $$\mathrm{\% Acetyl }=\frac{\left[\left({\mathrm{V}}_{\mathrm{B}}-{\mathrm{V}}_{\mathrm{S}}\right)\mathrm{ x Mx }43\right]}{\mathrm{m}}\mathrm{x }100 $$

where, V_*B*_ is the volume of HCl (mL) accustomed to titrate the blank sample, V_*S*_ is the volume of HCl (mL) applied to titrate the ISC sample, M is the concentration of the HCl (mol.L^−1^), 43 is the molar mass of the acetyl group (g.mol^−1^), and m is the mass (g) of the ISC [22]. The degree of substitution was calculated according to Eq. 2.

2 $$\mathrm{DS}=\frac{(162\mathrm{ x \% Acetyl })}{\left[43\mathrm{ x }100-\left(\left(43-1\right)\mathrm{x \% Acetyl }\right)\right]} $$

where 162 is the molecular weight of the anhydroglucose unit, 1 is the hydrogen mass. As the average of 3 intervals, the applied equations revealed that DS was about 0.1 of fabricated ISC particles.

### Corncob pretreatment for cellulose nanocrystals manufacture

The dried crushed corncob was dipped in a 10% NaOH solution (effective alkali; E.A.) with a solid/liquid ratio of 1:10 (wt/v) in an ultrasonicator water bath for about 30 min, then heated to 100 °C for 2 h. The cooking liquor was strained with a nylon cloth to extract the cellulosic pulp and fully washed with distilled water to eliminate any residual lignin from the pulp. The pulp was washed with deionized water and air dried. The partially delignified pulp resulted from an alkaline treatment was bleached by adding 20 mL of sodium hypochlorite (5%) at a temperature of 70 °C several times to dissolve the residual lignin and hemicellulose, giving a white cellulose pulp. The produced material was purified with hot deionized water and dried in an air oven at 60 °C.

Cellulose nanocrystals (CNCs) were produced by hydrolyzing bleached cellulose with 64% H_2_SO_4_ solution in a 1:10 (wt/v) ratio (cellulose: dilute H_2_SO_4_) at 55 °C for 65 min with vigorous and steady mechanical agitation. The hydrolysis reaction was squashed in an ice bath. The retrieved material was rinsed with distilled water and dialyzed against distilled water to attain a neutral pH. It was homogenized for 10 min in an ultrasonic water bath before freeze-drying and labelled as CNCs [23].

The cellulosic pulp fibers were located in a sealed glass jar comprising iodine crystals. The pot of iodine was placed in the convection oven for 15 min at 100 °C. The system was cooled to room temperature, before the fibers could be detached [20]. The iodinated extracted CNCs were exposed to oxidative thermal stabilization at 230 °C for 1 h in a muffle furnace, resulting in dark brown fibers that were reported as stabilized iodinated cellulose nanocrystals (SICNCs).

### Production of composite films based on thermoplastic starch (TPS)

The strategy of thermoplastic starch (TPS) films has been described in the following steps. Starch films were prepared by the casting process. About 5 g of S was mixed with 2.5 g of urea plasticizer diluted in 2 mL of distilled water and sonicated for 30 min to obtain a composite solution. The ratio of S to urea was kept constant at (1: 0.5) (wt/wt). The mixed solution was stirred continuously, employing a mechanical stirrer, and melted at a temperature of 80 °C for complete gelatinization. After the solution became viscous, it was poured into a petri-dish and manually pressed to obtain films of uniform thickness. The produced film was desiccated in an artificial air oven at 80 °C for 3 h. The prepared urea thermoplastic starch film was denoted as UTPS and was held at a temperature of 25 °C and preconditioned at a relative humidity of 60% until completely drying prior to testing.

The solution cast film from UTPS/ISC was fabricated in the same manner mentioned above with the addition of ISC as a filler in various concentrations of (2, 4, and 6%) (*wt/wt*) on a dry basis of corn starch by ultrasonic mixing. The mixture solutions were melted under continuous stirring at a temperature of 80 °C for complete gelatinization. The films were generated and designated as UTPS/ISC_2_, UTPS/ISC_4_ and UTPS/ISC_6_.

To fabricate UTPS/ISC_2_ grafted with SICNCs, we tended to follow the same technique as aforementioned with some modifications, which is the addition of ISC at a concentration of 2% (wt/wt) based on dry corn starch, then blending the mixture with SICNCs at the same concentration of 2% (wt/wt) based on dry corn starch. Starch-based film produced was denoted as UTPS/ISC_2_/SICNCs.

## Characterization methods

### Water barrier studies

#### Water vapor absorption study

The absorption of water vapor was measured based on the moisture absorption technique. Moisture absorption examination was performed according to the application of the following approach, each film was cut into 2 × 2 cm squares, then weighed and hung from a wire. The wire was suspended in a beaker containing evaporated water at 100 °C. Water vapor release was achieved by varying the weight of each sample [S, UTPS, UTPS/ISC_2_, UTPS/ISC_4_, UTPS/ISC_6_, and UTPS/ISC_2_/SICNCs]. Every half hour, each sample was weighed for 3 h. Using the following equation the mean value of the water vapor absorption was calculated from three intervals:

3 $$\mathrm{Water\,vapor\,release }\left(\mathrm{\%}\right)=\frac{{W}_{t}-{W}_{o}}{{W}_{o}} x100$$

where W_t_ is the weight of the sample after "t" time and W_º_ is the initial weight of the sample film. The relative humidity resulted from the absorption of saturated moisture produced by water vapor at 100 °C was ≈ 95%.

#### Water solubility study

The water solubility of each film was determined using the dry matter percentage of the soluble film after immersion in distilled water. The fabricated samples were cut and dried at 100 °C to determine the dry weight of the material. Each sample was sealed in a 40 mL beaker of distilled water and incubated for 2 h at 25 °C with periodic delicate shaking. The samples were removed from water by filtration and insoluble dry matter was determined by drying in an oven at 100 °C up to a constant mass [24]. The percentage of soluble matter (% SM) was predetermined as the average of 3 interval times using the following equation:

4 $$\mathrm{SM}\left(\mathrm{\%}\right)=\frac{\mathrm{ initial\,dry\,weight }-\mathrm{ final\,dry\,weight}}{\mathrm{initial\,dry\,weight}} \times 100$$

### Fourier transform infrared spectroscopy

The influence of plasticization and modulation on the functionality of the surface of starch-based films was analyzed through FT-IR spectroscopy employing the KBr pellet approach on an FT-IR NICOLET 8700 spectrometer (Thermo Scientific, Loughborough, United Kingdom) in the spectral range of 400–4000 cm^−1^ along with four resolutions averaged over 40 scans.

### Scanning electron microscopy

The surface morphologies of the fabricated composite film samples were checked by applying QUANTA FEG 250 ESEM (Japan).

### Surface area measurements

The specific surface area (Brunauer–Emmett–Teller (BET) method) and the features of the pores of the synthesized composite films were evaluated by adsorption–desorption of N_2_ at 77K with a surface area analyzer model (Quanta Chrome Instruments, NOVA Automated GAS Sorption System Version 1.12, USA).

### Liquid phase adsorption characteristics

The aim of this work is to study the possibility of treating aqueous solutions contaminated with crystal violet dye (CV) by UTPS, UTPS/ISC_2_, and UTPS/ISC_2_/SICNCs composite films. Consequently, stock solutions with diverse concentrations were prepared by dissolving (10–100) mg of CV dye in 1 L of distilled water. 100 mg of fabricated samples were administered at pH (6.5) using 0.1 M HCl and 0.1 M NaOH with 10 mL of CV dye solution. To attain equilibrium, every sample was kept in a rotating shaker (IKA KS 130 shaker with 230 V-50/60 Hz power supply, basic stirrer and room temperature range of 5 to 50 degrees with numerical LCD speed & time display) at 220 r.p.m, for 24 h at room temperature. Preliminary tests showed that 24 h was adequate to reach equilibrium. The UV–visible absorption spectra of the supernatant solution were analyzed employing a UV–visible spectrophotometer (Type UV-2401PC) in a 1 cm quartz cuvette to check the characteristic absorption peaks of CV dye at a wavelength of 590 nm. The equilibrium adsorption amount, q_e_ (mg/g), was determined according to the following equation:

5 $${\mathrm{q}}_{\mathrm{e}} =\frac{\mathrm{V }\left({\mathrm{C}}_{\mathrm{o}}-{\mathrm{C}}_{\mathrm{e}}\right)}{\mathrm{m}} $$

where C_o_ and C_e_ (mg/L) are the initial and equilibrium concentrations in the liquid phase, V (L) is the volume of the equilibrium solution, and m (g) is the mass of the adsorbent. The percentage of dye removal from the aqueous solution was conditioned by applying the following equation, where R is the removal efficiency of the dye:

6 $$R (\%) =\frac{ ({C}_{o}-{C}_{e})}{{C}_{o}} $$

The Langmuir isotherm is used to valuate the coverage of a monolayer with uniform adsorption energies onto the surface without adsorbate transmigration into the surface plane [25, 26]. It can be described as the following equation:

7 $$\frac{{\mathrm{C}}_{\mathrm{e}}}{{\mathrm{q}}_{\mathrm{e}}}=\frac{1}{{\mathrm{K}}_{\mathrm{L}}{.\mathrm{q}}_{\mathrm{m}}}+ \frac{{\mathrm{C}}_{\mathrm{e}}}{{\mathrm{q}}_{\mathrm{m}}} $$

where C_e_ is the equilibrium dye concentration (mg/L), q_e_ is the adsorption capacity at equilibrium (mg/g), q_m_ is the maximum adsorption capacity related to complete monolayer coverage on the surface (mg/g); and K_L_ is the Langmuir constant (L/mg), indicating the nature of adsorption. The main feature of the Langmuir isotherm can be stated in terms of the equilibrium parameter R_L_, which is the dimensionless constant known as the separation factor [27]. C_o_ is the initial concentration for maximum removal efficiency.

8 $${\mathrm{R}}_{\mathrm{L}} =\frac{1}{\left(1 + {\mathrm{K}}_{\mathrm{L}}.{\mathrm{C}}_{\mathrm{o}}\right)} $$

The R_L_ value indicates whether the adsorption feature is favorable if 0 < R_L_ ≤ 1, unfavorable if R_L_ > 1, linear if R_L_ = 1, and irreversible if R_L_ = 0 [27, 28].

The Freundlich isotherm estimates the adsorption properties of the heterogeneous surface [29, 30]. It can be expressed as the following equation:

9 $${\mathrm{q}}_{\mathrm{e}}={\mathrm{K}}_{\mathrm{F}}.{\mathrm{C}}_{\mathrm{e}}^{\frac{1}{\mathrm{n}}}$$

where K_F_ (mg/g)(L/mg)^1/n^ represents the Freundlich isotherm constant, and n represents the adsorption intensity constant. At n = 1, the separation between the two phases is independent on the concentration. If the value of 1/n is < 1, this indicates that the adsorption is normal. In contrast, 1/n > 1 demonstrates cooperative adsorption [31].

The Temkin isotherm equation presumes that the heat of removal of full molecules in the layer could decrease linearly instead of logarithmically with coverage due to adsorbent-adsorbate interactions. This model is given as:

10 $${\mathrm{q}}_{\mathrm{e}}={\mathrm{B\,ln\,K}}_{\mathrm{T}}+{\mathrm{B\,ln\,C}}_{\mathrm{e}} $$

11 $$\mathrm{B}=\frac{\mathrm{RT}}{{\mathrm{b}}_{\mathrm{T}}} $$

where K_T_ is the equilibrium constant of the Temkin isotherm (L/mg) related to the maximum binding energy, b_T_ is the Temkin isotherm constant corresponding to the heat of adsorption (J/mol), R is the constant of universal gas (8.314J/mol.K), and T is the temperature (K).

Dubinin-Radushkevich assesses both homogeneous and heterogeneous surfaces with a Gaussian energy distribution. It features a compelling specific parameter for the average free energy, which is utilized to differentiate physical and chemical adsorption. The equation is given as follows:

12 $${\mathrm{ln\,q}}_{\mathrm{e}}={\mathrm{ln\,q}}_{\mathrm{mDR}}-{\mathrm{K}}_{\mathrm{DR}} . {\upvarepsilon }^{2 } $$

13 $$\upvarepsilon =\mathrm{RT\,ln}\left(1+ \frac{1}{{\mathrm{C}}_{\mathrm{e}}}\right)$$

where q_mDR_ (mg/g) is the adsorption capacity of saturation theory (mg/g), K_DR_ (mol^2^/J^2^) is the Dubinin-Radushkevich isotherm constant, and ε is the potential Polaniyi (J/mol). This model is used to differentiate physical and chemical adsorption through its average free energy, E (J/mol), which is required to remove an adsorbate molecule from its position at the adsorption site to infinity. It is calculated using the equation:

14 $$\mathrm{E}=\frac{1}{\sqrt{2{\mathrm{K}}_{\mathrm{DR}}}} $$

### Mechanical performance

Uniaxial tensile examination with a comprehensive (LR10K; L1oyd Instruments, Fareham, UK) machine has been upgraded to UTPS, UTPS/ISC_2_, and UTPS/ISC_2_/SICNCs composite films. The tensile test of the samples was carried out in a humid environment for 5 days. The fiber mats (30 mm length × 10 mm width × 2 mm breadth) were adhered to a paper fixing tab with an epoxy adhesive with a gauge length of 20 mm and a tensile speed of 2.5 mm/min. When the sample was firmly gripped and cut from both sides of the tab, the tensile test began. The mean values of tensile strength, modulus of elasticity, and elongation at break were estimated at least 3 times for each sample [32]. The tensile stress and strain of the tested samples were calculated by applying the following equations:

15 $$\upsigma =\frac{\mathrm{F}}{\mathrm{A}} $$

where σ is the stress (N/mm^2^ or MPa), F is the load (N), and A is the cross-sectional area (mm^2^).

16 $$\upvarepsilon =\frac{\left(\mathrm{L}- {\mathrm{L}}_{\mathrm{o}}\right)}{{\mathrm{L}}_{\mathrm{o}}}$$

where ε is the strain, L_◦_ is the actual sample length (mm), and L is the sample length at breaking point (mm). The modulus of elasticity, E, is a measure of the ability of a material to tolerate longitudinal changes under tension or compression. It can be represented by a linear regression analysis of the initial linear section of the stress–strain curves, applying the formula illustrated as follows:

17 $$\mathrm{E}= \frac{\mathrm{Stress}}{\mathrm{Strain}}=\frac{\mathrm{\Delta \sigma }}{\mathrm{\Delta \varepsilon }}$$

where *E* is Young’s modulus (MPa), and tensile strength can be determined from the maximum stress point (σ_max_, MPa).

## Results and discussion

### Water barrier behavior study

#### Water vapor absorption

**Fig. 1:**
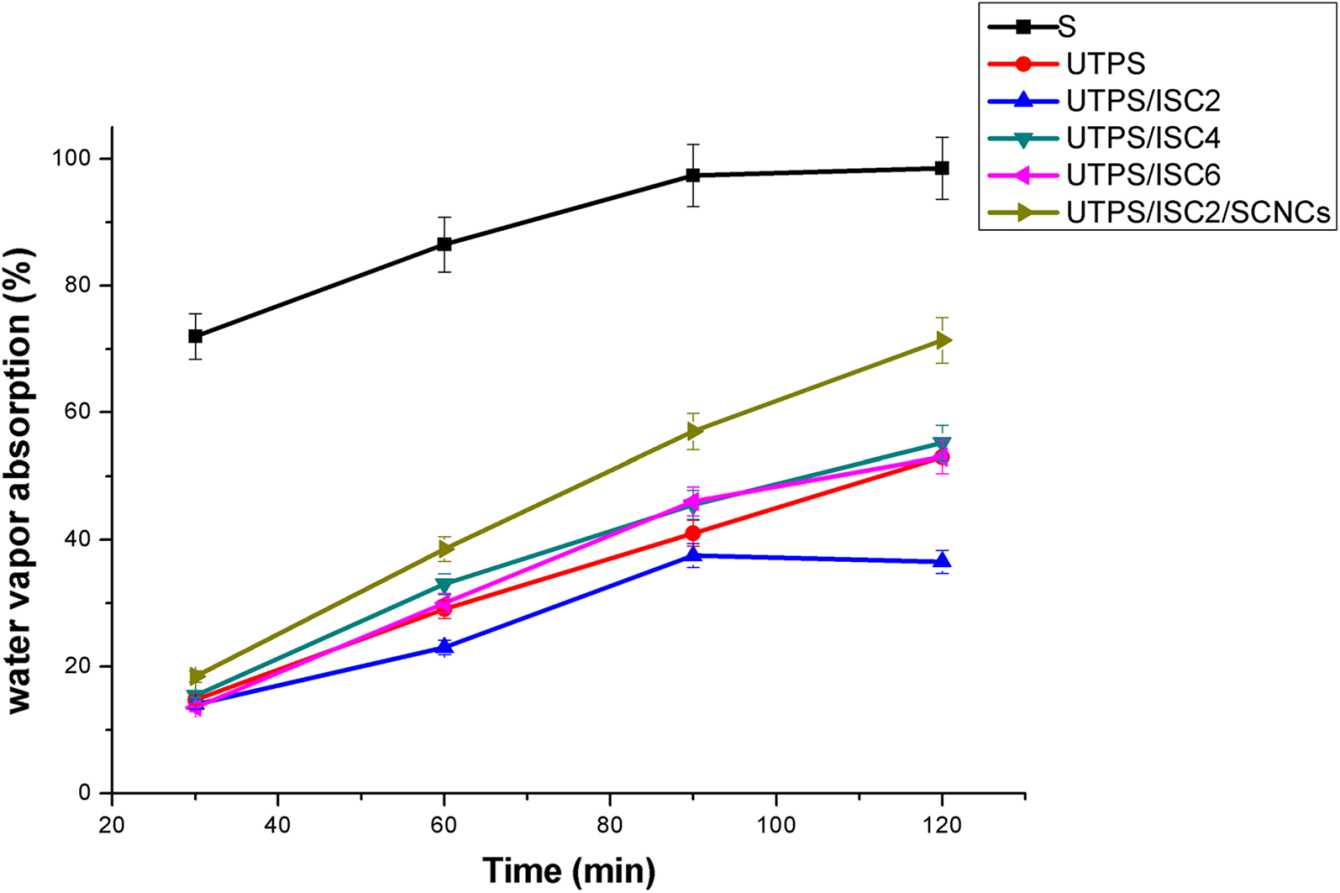
Water vapor absorption profile of starch-based composite films. Bars represent the standard error of the mean (n = 3)

By assessing the amount of water vapor that can pass through the pores and boundary surface of the bio-polymer film, the experimental study on the water vapor absorption aids to understand the barrier behavior of as-synthesized films [33]. Table 1 and Fig. 1 demonstrate the rate (%) of water vapor of starch-based films under various processing parameters throughout time. It is obvious from Table 1. that water vapor absorption varied for S from 72 to 98.5%, whereas the presented value by UTPS was between 14.7 to 53%. Those based on the inclusion of ISC such as UTPS/ISC_2_, UTPS/ISC_4_ and UTPS/ISC_6_ ranged from 14 to 36.5%, from 15.4 to 55.2% and from 13.5 to 53% respectively, UTPS/ISC_2_ had the lowest amount of the water vapor absorption. From Fig. 1, a high gradient was observed for water vapor absorption of starch due to its strong hydrophilicity, while it showed a moderate gradient in UTPS and UTPS/ISC_2_ implying a more controlled absorption property as a result of plasticization and the modification of ISC. This phenomenon was explained by the presence of urea that enfolds the internal polymeric chains of the starch increasing the intermolecular forces between the polymer chains and decreasing the free volume, hence it can prevent water molecules from spreading through the films. Furthermore, the hydrophobic ester groups in the cross-linked starch citrate introduced new intra-molecular bonds that limited the amount of water vapor that can be absorbed in starch [34], so it can be very useful in the preservation of fruits and vegetables due to the fact that it does not absorb the essential water content of them. Meanwhile, the incorporation of SICNCs into UTPS/ISC_2_ raised the water vapor (%) to 71.4 after 2 h revealing their poor release profile. This was assumed to be related to the high hydrophilicity and porosity of SICNCs, which favored the absorption of water molecules and contributed to an increase in the water vapor permeability of the film. Most non-absorbency applications are impeded by poor barrier properties of this composite film, however low water vapor absorption films have better barrier properties and good packaging applications [35].

**Table 1:** Water barrier characteristics for the prepared starch-based composite films

WVR (%)					Water solubility (%)
Specimens	30 min	60 min	90 min	120 min	120 min
S	72	86.5	97.4	98.5	14.5
UTPS	14.7	29	41	53	11.6
UTPS/ISC_2_	14	23	37.5	36.5	9.8
UTPS/ISC_4_	15.4	33	45.5	55.2	24
UTPS/ISC_6_	13.5	30	46	53	22
UTPS/ISC_2_/SICNCs	18.4	38.5	57	71.4	Insoluble

#### Water solubility

**Fig. 2:**
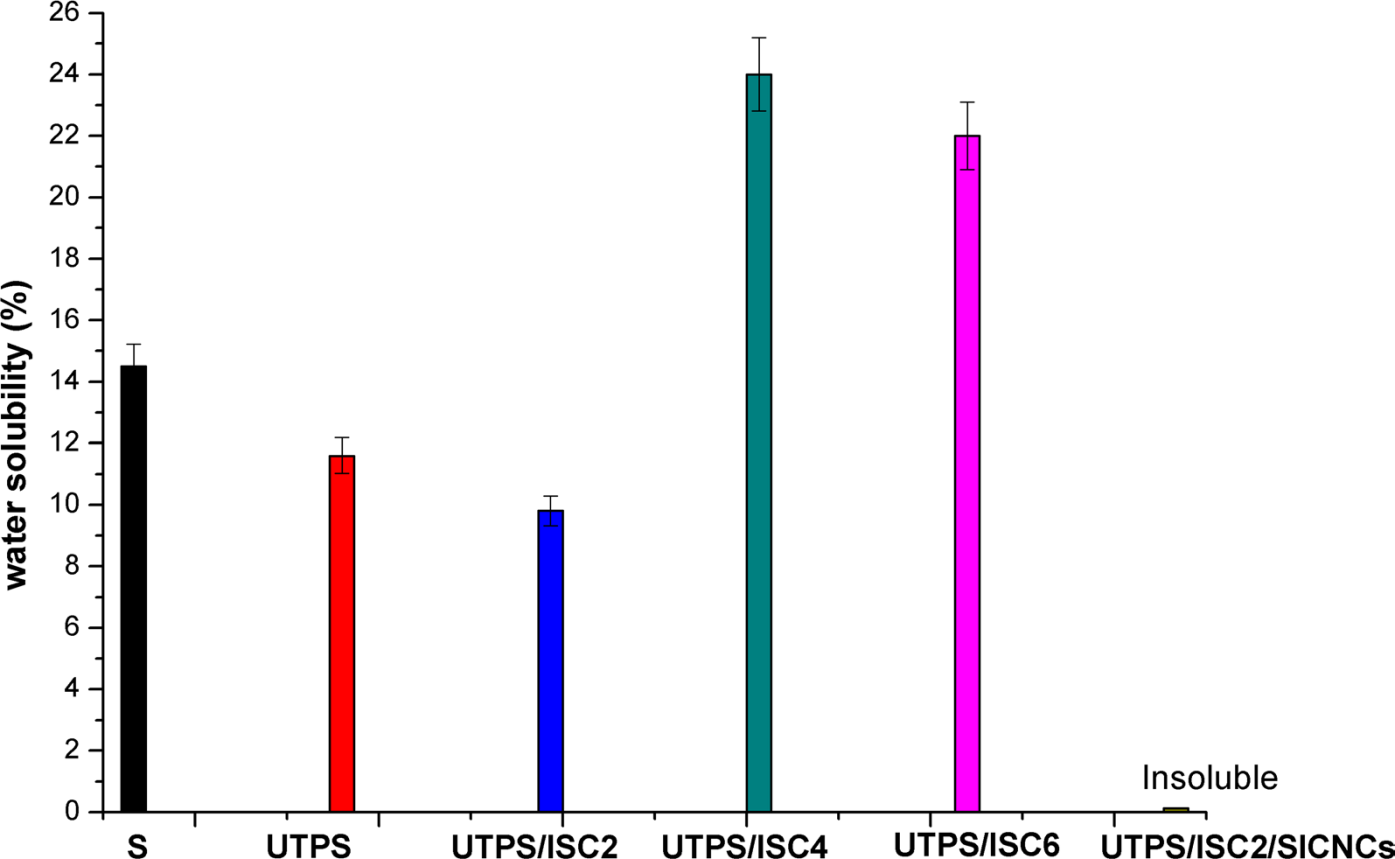
Water solubility (%) of starch-based composite films. Bars represent the standard error of the mean (n = 3)

The water solubility of films is one of the most important properties for understanding biodegradable packaging. Starch applications are generally limited because they are unstable with changes in temperature, water, pH, and shear forces [36]. Water insolubility is useful for various purposes, including improving product quality and creating water barriers to keep food safe. It is recognized that water solubility is the measure of a material’s resistance to water. On the other hand, the solubility in water demonstrated degradation behavior of the prepared starch-based composite films when disposed of in water at room temperature [37]. According to Table 1 and Fig. 2, UTPS revealed lower solubility (11.6%) than S-film (14.5%). Since amylose and amylopectin, which are found in native starch, regulate starch solubility in different ways [38]. Furthermore, blending urea with hydrolyzed starch short chains reduced the product solubility of UTPS-based film because the existence of –NH groups in urea caused the formation of hydrogen bonds with hydroxyl groups in starch chains, making the film more hydrophobic. The solubility of UTPS decreased from 11.6 to 9.8% with the incorporation of ISC_2_. This result revealed that the modification of starch with urea and cross-linking (ISC_2_) strengthened the bonds between adjacent starch chains, thereby reducing their tendency to split during solubility [39]. Ester groups of citric acid protected the degradation of starch and reduced its hydrophilicity with water according to the conclusion of a water vapor absorption study [40]. Furthermore, it is reported that as the ISC content increased, the solubility (%) increased. This can be attributed to the large number of short-chain amyloses formed as a result of acid hydrolysis during the esterification of starch, which readily dissociated and diffused from ISC upon swelling and dissolution [40].

Surprisingly, UTPS/ISC_2_/SICNCs revealed insoluble properties and no water absorption at room temperature, owing to their rigidity against water solubility as measured and the creation of a three-dimensional network of cellulose via the generated hydrogen bond between starch and SICNCs. This network limited the solubility of the polymer, caused strengthening of the network, and reduced the contact between water and the produced film. The results highlighted the composite films resistance to humidity, temperature, and water solubility.

### FT-IR spectroscopy

**Fig. 3:**
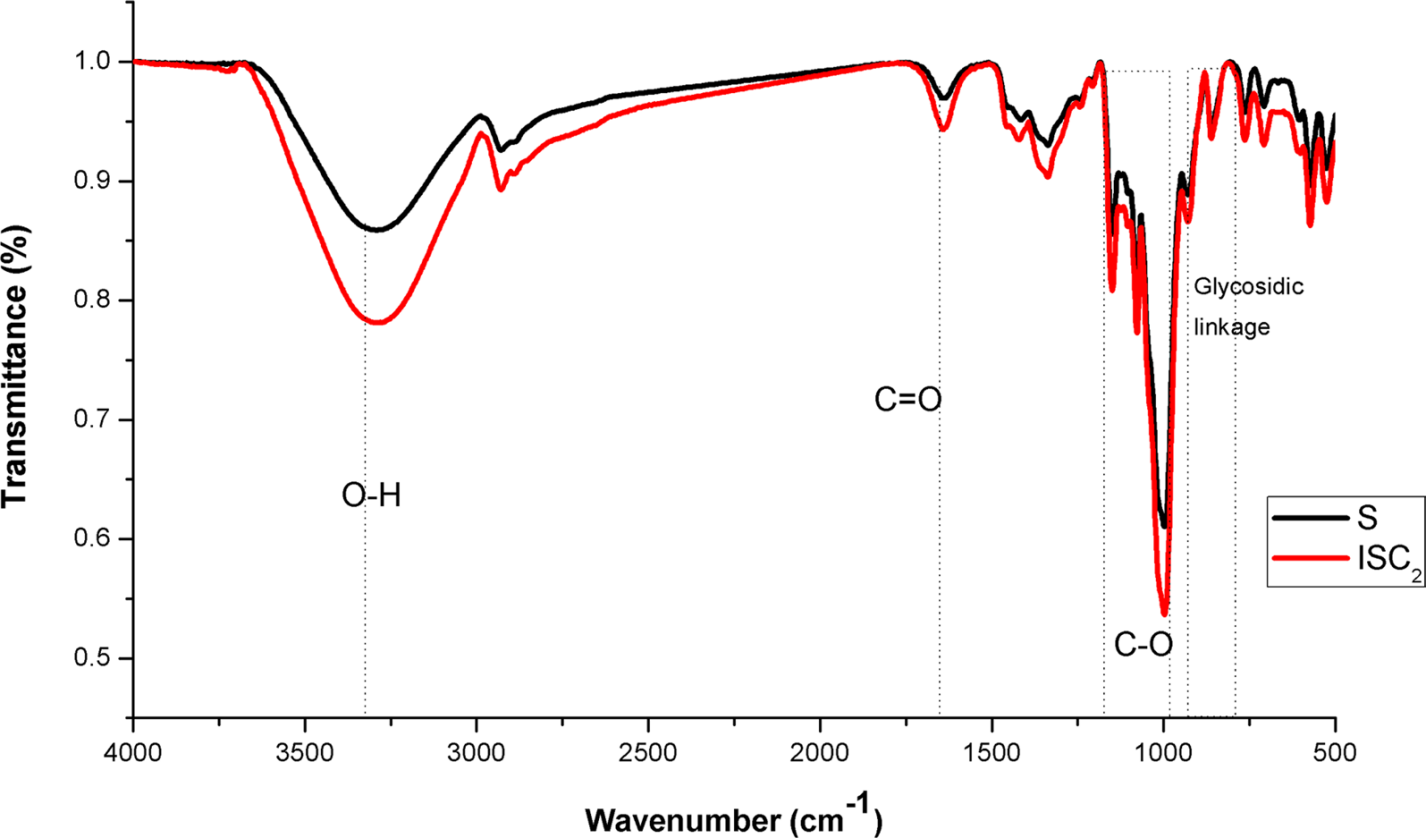
FT-IR spectra of native starch and its modified citrate (ISC)

The introduction of extra functional groups into the starch-based films was validated by FT-IR spectroscopy. The spectra of native starch (S) and ISC_2_ are revealed in Fig. 3. Peaks in the S spectrum can be seen at 1020–1250 cm^−1^. The C-O stretching vibration band has been assigned to these peaks. A peak at 1650 cm^−1^ was attributed to tightly bound water by a strong H-bond, and the starch biopolymer’s characteristic absorption bands were in the range of 2800–3000 cm^−1^ due to the -CH stretching of starch moieties. Another exceedingly broad band at 3292 cm^−1^ was associated with the vibration of the linked hydroxyl group (O–H) [41], whereas the skeletal mode of the glycosidic linkage was observed at around 1000 cm^−1^. ISC_2_ showed similar FT-IR spectra with increasing intensity of the characteristic peaks at 1650 cm^−1^ and 1020–1150 cm^−1^ owing to carbonyl group generation (C = O), and C-O stretching vibration, respectively [42]. The band in the region 1376 cm^−1^ of ISC_2_ was relevant to the stretching of the –CH_3_ of acetyl groups. The expansion in the band intensity of ISC_2_ has been attributed to new citrate functional groups or to new intramolecular hydrogen bonds among the hydroxyl groups concerning the starch and the carbonyl group regarding citrate.

**Fig. 4:**
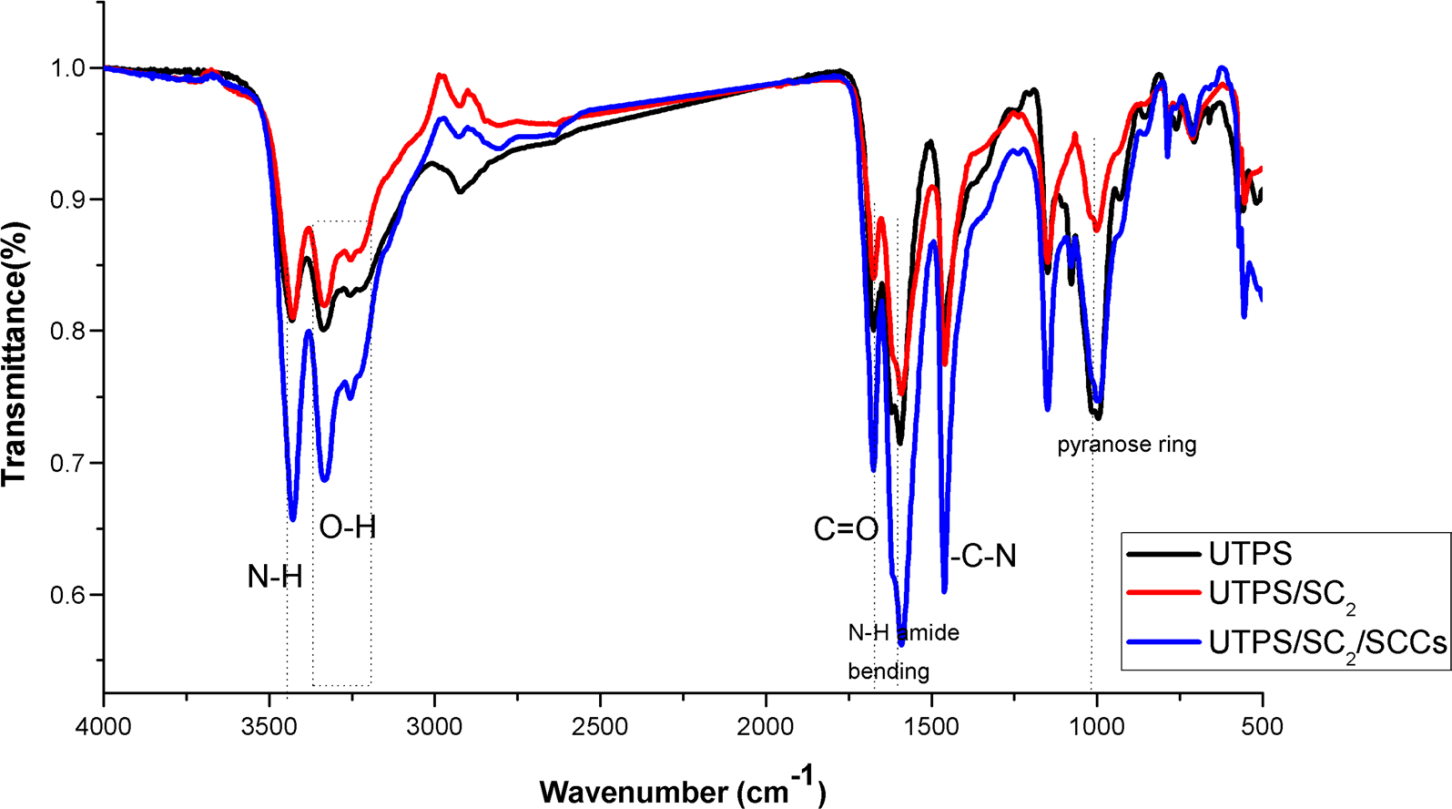
FT-IR spectra of as-synthesized starch-based composite films

The FT-IR spectra for UTPS, UTPS/ISC_2_ and UTPS/ISC_2_/SICNCs are displayed in Fig. 4. The presence of intermolecular and intramolecular hydrogen bonds in the polymer matrix was suggested by the appearance of two-featured absorption bands in the range of 3260 and 3335 cm^−1^ in the UTPS spectra, represented by the OH vibration. Urea has a distinct absorption band in the regions at 3432 cm^−1^ which is characteristic of N–H amide stretching, and N–H amide bending with characteristic peaks found at 1595 cm^−1^ and 1621 cm^−1^, respectively [43]. Furthermore, the absorption peak located near 1463 cm^−1^ showed the strong stretching of the -C-N groups, this peak emerged sharper with the incorporation of ISC_2_ in the UTPS/ISC_2_ and UTPS/ISC_2_/SICNCs spectra, which explained the association of ISC_2_ in the starch plasticizing system [3]. The absorption band derived from the −C = O stretching vibration group of urea at wavenumber 1675 cm^−1^ appeared sharper after incorporation of ISC_2_ supporting its contribution to the starch chains and urea. The spectra of UTPS/ISC_2_ and UTPS/ISC_2_/SICNCs depict the typical bands of urea and starch citrate, for a peak around 2800 cm^−1^ that indicates the C-H stretching of an aliphatic methyl group with a noticeable reduction in its intensity, suggesting new elongation of the backbone chain after mutation with ISC_2_ and SICNCs. For UTPS/ISC_2_/SICNCs, the absorption peak at 3335 cm^−1^ became slightly sharper for the hydroxyl group due to the hydrogen bond interaction when starch and SICNCs were included together in the nanocomposite manufacture. With the absence of the functional groups of lignin and hemicellulose, the characteristic band of the cellulose pyranose ring was evident at 1070 cm^−1^, ensuring the efficiency of the delignification and bleaching pretreatment steps [44, 45]. The distinct functional groups of the N–H, O–H, C–H, C = O and β–glycosidic linkage appeared with greater intensity, indicating that the cellulose adhered to the starch-based composite films had a rigid structure [46].

### SEM analysis

**Fig. 5:**
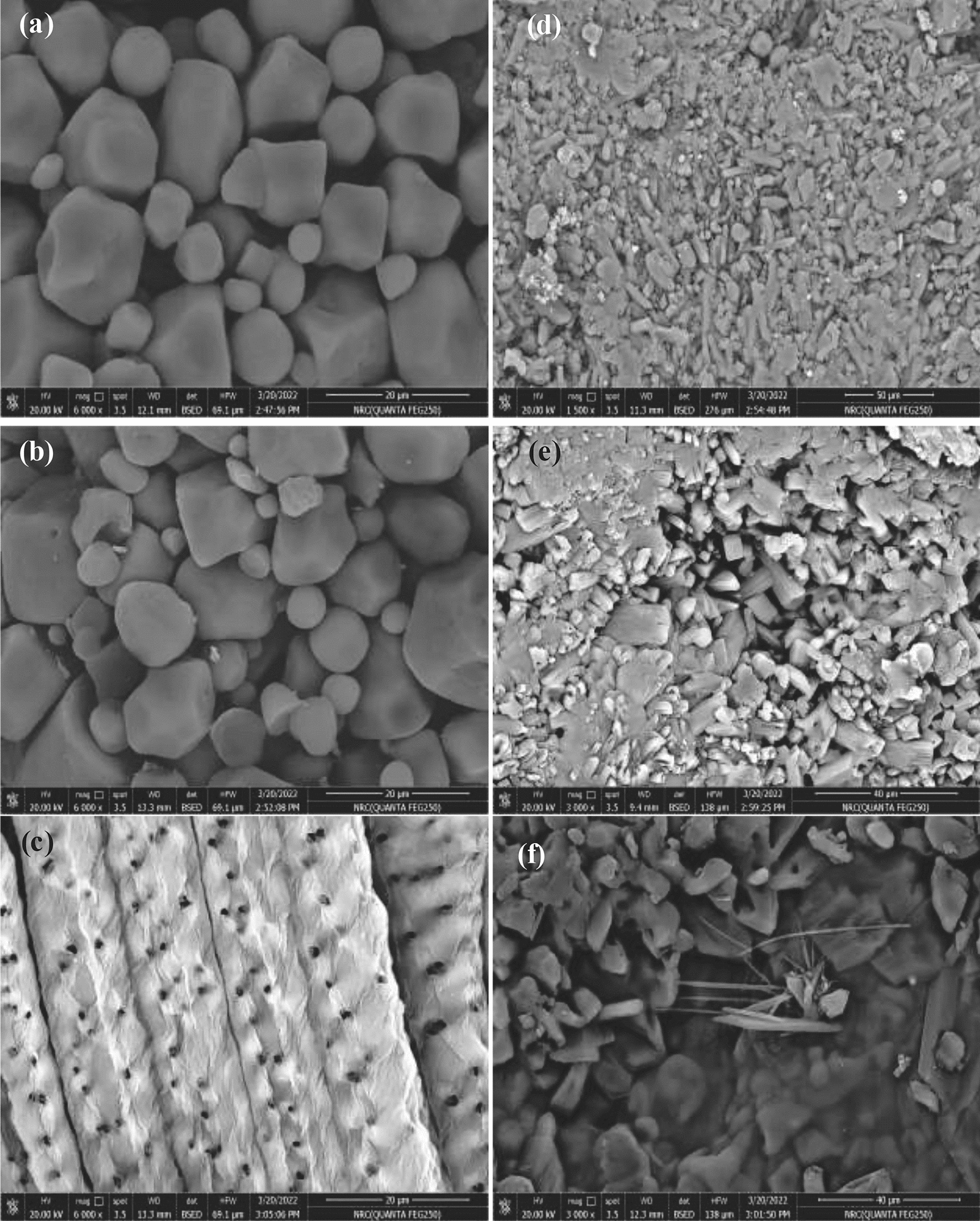
SEM micrographs of **a** S **b** ISC **c** SICNCs **d** UTPS **e** UTPS/ISC_2_ and **f** UTPS/ISC_2_/SICNCs composite films

Figure 5a, b reveals the SEM micrographs of native and acetylated corn starch (ISC). Corn starch granules are polygonal and spherical with random particles that could easily agglomerate and stack. The granules ranged in size from 8 to 12 µm. The ISC_2_ particles still persist intact with the same particle size and shape, but with wrinkled cores and lengthy tortuous grooves that may be attributable to the exposure to iodine and citric acid. Iodine, acting as an oxidizing agent, caused defects on the surface of the starch, making it rougher, while citric acid induced eco-corrosion on the starch surface. The minor change in the micrographs was assumed to the increase of D.S which may damage the intermolecular hydrogen bonds and reduce the resilience of starch, as interpreted by Sha et al. [47]. As demonstrated by the SEM micrograph of SICNCs in Fig. 5c, it showed porous bundles of fibers arranged vertically and firmly enclosed in a regular shape which increased the diameter of the outer surface (12 μm) and the pore diameter (1 μm).

The micrographs of starch-based composite films are present in Fig. 5d–f. When starch was heated in presence of a plasticizer of UTPS, the starch grains were destroyed to form a continuous phase of small fragments in the form of a smaller diameter rod in range (600 nm–2.7 μm). The presence of plasticizer caused the breakage of intra- and intermolecular hydrogen bonds between starch polymer chains, resulting hydrogen bonds inside the starch granules. This made native starch plastic at temperature conditions [48, 49], as reflected in Fig. 5d. The micrograph of UTPS/ISC_2_ in Fig. 5e displayed small dense particles that were tightly agglomerated with starch particles which were considered to be intertwined like cubes with a rough surface, as a consequence the crystallinity of starch granules was reduced and the plasticizing impact was enhanced [50]. This validated the effect of ISC_2_ that caused chain fragmentation of starch molecules. Thus, it decreased the interaction between the molecular chains of the starch improving the plasticization process of starch [51]. Nevertheless, the combined effect of SICNCs in the UTPS/ISC_2_ film observed in Fig. 5f manifested that the starch granules were largely fragmented into a rougher surface shape, confirming that SICNCs promoted starch plasticization [52]. Owing to the reactive surface of SICNCs and the strong adhesion between the SICNCs and the polymer matrix by hydrogen bonding, it may be able to provide a good dispersion matrix of smaller SICNCs with a diameter ranging (500–900 nm).

### Porous texture of starch-based films

Specific surface area evaluation (S_BET_, m^2^/g), total pore volume (V_t_, cc/g), and mean pore diameter (D_p_, nm) were evaluated for the starch-based films according to the Brunauer–Emmett–Teller (BET) equation, and the data are given in Table 2.

**Table 2:** Textural characteristics of the prepared starch-based composite films

Fabricated samples	S_BET_ (m^2^/g)	V_t_ (cc/g)	D_p_ (nm)
UTPS	0.14	6.4 × 10^–5^	1.9
UTPS/ISC_2_	0.1	3.3 × 10^–5^	1.44
UTPS/ISC_2_/SICNCs	0.05	6.7 × 10^–7^	0.05

Interestingly, the surface area and total pore volume of UTPS, UTPS/ISC_2_ and UTPS/ISC_2_/SICNCs were (0.14 m^2^/g, 6.4 × 10^–5^ cm^3^/g), (0.1 m^2^/g, 3.3 × 10^–5^ cm^3^/g) and (0.05 m^2^/g, 6.7 × 10^–7^ cm^3^/g), respectively representing the inhibitory effect of ISC_2_ and SICNCs on UTPS assembly. With respect to the average pore diameter, the results revealed that the produced starch-based films were consistent with a microporous nature. The reduction of microporosity and S_BET_ with the exfoliation of ISC_2_ and SICNCs in the polymer matrix confirmed their effective involvement in enhancing the plasticization of starch-based films, as addressed in SEM micrographs.

### Adsorption equilibrium and isotherm models

**Fig. 6:**
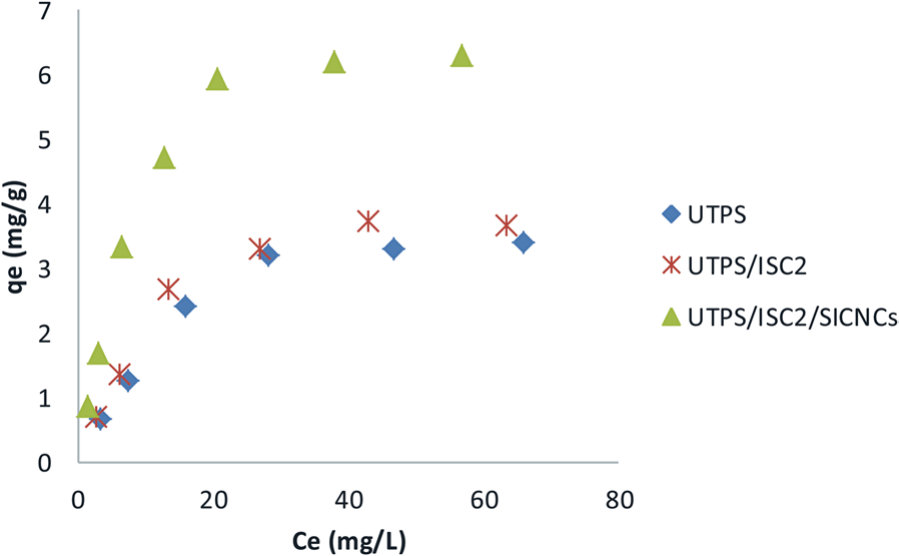
Adsorption isotherm plateau for CV dye uptake on as-synthesized starch-based composite films

**Fig. 7:**
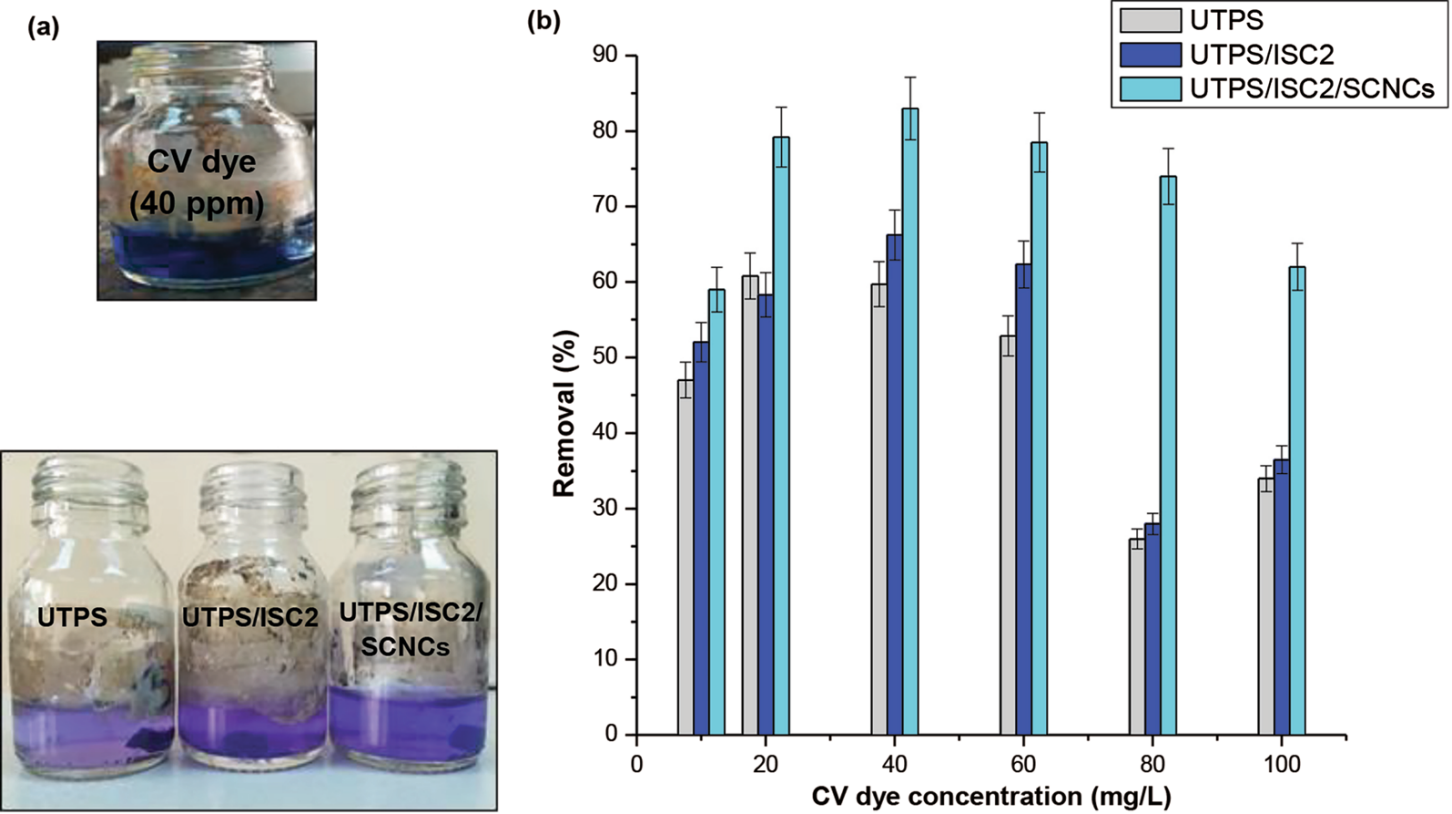
**a** Photographs of the CV dye solution (40 mg/L) before and after adsorption **b** Effect of the adsorbate concentration on dye removal (%).Bars represent the standard error of the mean (n = 3)

As shown in Fig. 6, the adsorption isotherm is useful for characterizing how the adsorbate interacts with the adsorbent and estimating adsorption capacity. Adsorption equilibrium studies were carried out in this work with initial crystal violet (CV) dye concentrations ranging between 10 and 100 mg/L. As seen in Fig. 6, when the initial dye concentration increased, the adsorption capacity of fabricated samples also linearly increased to a concentration of 40 mg/L, so a slow decrease was noted with a maximum removal (%) reaching 83% for UTPS/ISC_2_/SICNCs, as obvious in Table 3, whereas, the highest (%) for UTPS (60.8%) was recorded at the initial concentration (20 mg/L), meanwhile the highest rate (%) for UTPS/ISC_2_ (66.2%) was obtained at 40 mg/L. This phenomenon can be clarified by the certainty throughout the initial phases of adsorption, a significant number of free sites were available on the adsorbent surface, but these sites were eventually occupied by dye molecules and the adsorption capacity remained constant after equilibrium [53]. As established by Borhade and Kale [54], adsorption may be favored at lower initial dye concentrations when the adsorbent materials have a small surface area since the mass transfer forces are low. Consequently, it is reasonable to state that UTPS/ISC_2_/SICNCs film was more effective in remediating moderately concentrated CV from wastewater than other produced starch-based composite films. The adsorption of the CV dye on the manufactured starch-based composite films proceeded via electrostatic interactions with the highly negatively charged binding sites(FT-IR section), which was greatly enhanced after the incorporation of ISC_2_ and SICNCs into the UTPS film. Therefore, the adsorption of the CV dye was related to the surface chemistry rather than to the textural properties. The results of the percentage removal of CV by fabricated starch-based composite films are presented in Fig. 7. The percentage removal of fabricated samples onto CV dye was matched with diverse adsorbents from the publications and stated in Table 3.

**Table 3:** Comparison of our fabricated samples performance with various recorded adsorbents for the removal of CV dye

Fabricated samples	Q_m_ (mg/g)	C_◦_ (mg/L)	pH	Dose (g/L)	References
UTPS	4.4	40	6.5	10	This work
UTPS/ISC_2_	4.6	40	6.5	10	This work
UTPS/ISC_2_/SICNCs	7.6	40	6.5	10	This work
Waste coffee husk	1.38	12	3	1.5	[15]
Magnetic charcoal	10	40	8	1	[56]
Nanomagnetic iron oxide	12.5	5	7	0.5	[57]
Chitosan magnetic microspheres	28.2	-	7	0.1	[58]
Activated carbon from lemon	23	10	9	1.25	[59]

### Modeling of the adsorption isotherm

**Fig. 8:**
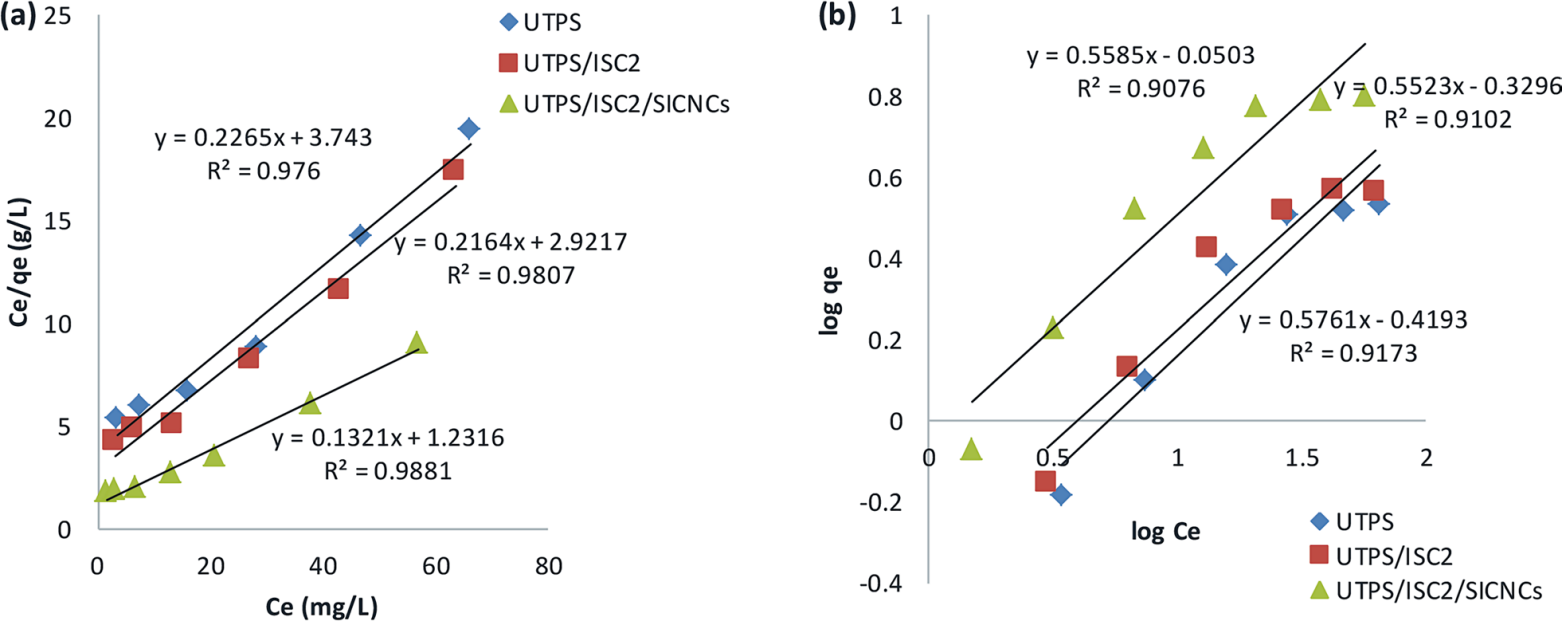
**a** Langmuir and **b** Freundlich isotherm models of starch-based composite films for CV dye adsorption

**Fig. 9:**
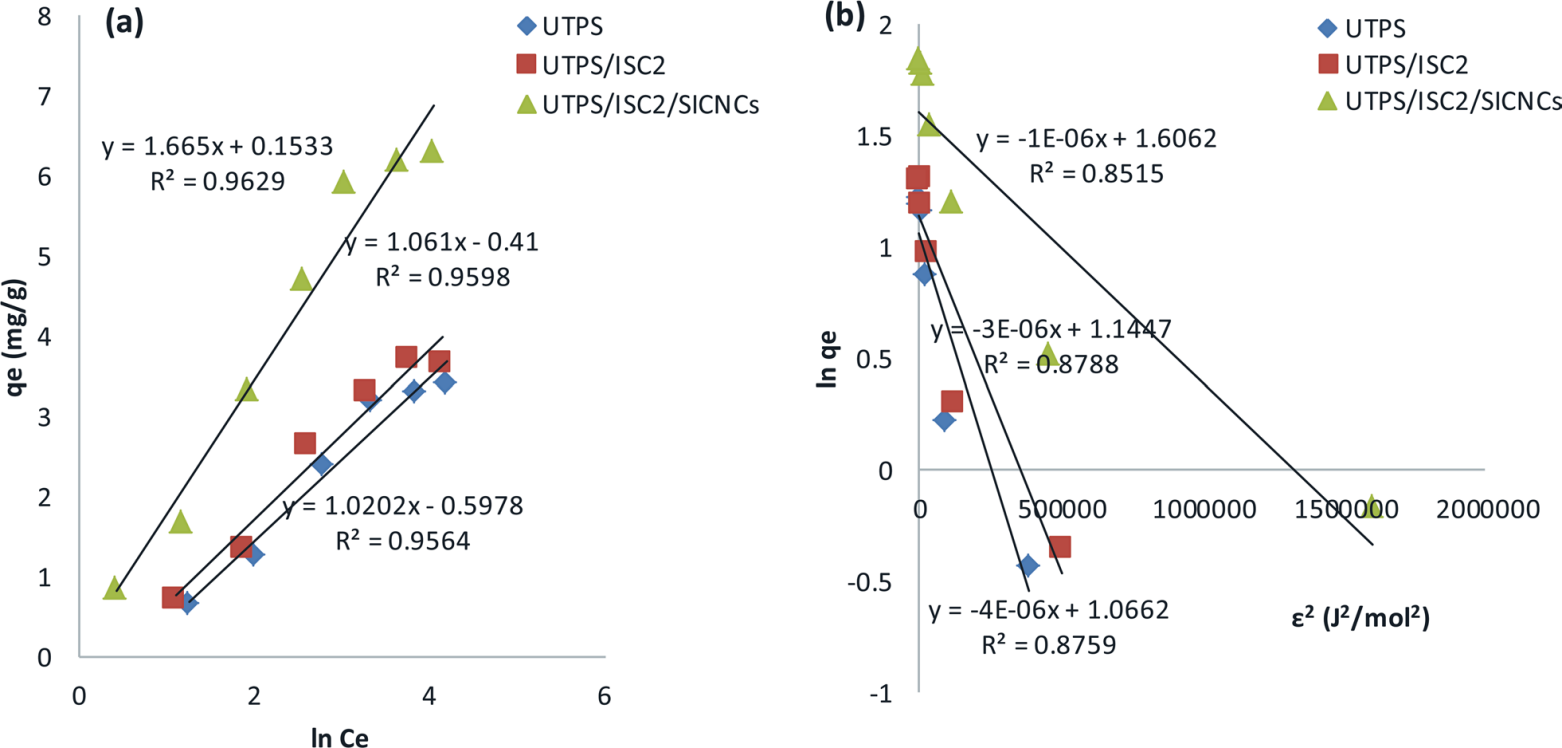
**a** Temkin and **b** Dubinin–Radushkevich isotherm models of starch-based composite films for CV dye adsorption

Some isotherm models have been utilized to evaluate the isotherm performance for CV adsorption and the fitting of the experimental data. Langmuir, Freundlich, Temkin, and Dubinin-Radushkevich were among them. In Figs. 8, 9, and Table 4, the plots of various adsorption models and fitting model parameters with R^2^ were displayed individually. Langmuir is more applicable than Freundlich, Temkin and Dubinin-Radushkevich models in terms of R^2^ values. The Langmuir model anticipated the establishment of a regular and homogeneous monolayer adsorbate on the outside surface of the adsorbent. Additionally, the R_L_ values were lower than 1, indicating that the dye was being adsorbed favorably. The values of n from the Freundlich parameters were greater than unity, indicating that the process of CV adsorption onto the fabricated samples was effective. The positive Temkin constant (b_T_) values ( 2.43, 2.34, and 1.5 J/mol) of UTPS, UTPS/ISC_2_, and UTPS/ISC_2_/SICNCs, respectively for the Temkin isotherm revealed that the adsorption was exothermic [55]. The values of mean free energy (E) for the Dubinin-Radushkevich (D-R) isotherm model for the adsorption of CV dye onto UTPS, UTPS/ISC_2_, and UTPS/ISC_2_/SICNCs are 0.353, 0.408, and 0.707 kJ/mol, respectively supporting the suggested dominating mechanism of physisorption [55]. Table 4 lists the values of these four sorption parameters. The data clearly displayed that the R^2^ values seemed to follow the order; Langmuir > Temkin > Freundlich > Dubinin-Radushkevich.

**Table 4:** Langmuir, Freundlich, Temkin, and Dubinin–Radushkevich isotherm constants for the adsorption of CV dye onto produced samples

Adsorbent	Langmuir isotherm model				Freundlich isotherm model			
	q_m_ (mg/g)	K_L_ (L/mg)	R_L_	R^2^	1/n	n	K_F_ (mg/g)(L/mg)^1/n^	R^2^
UTPS	4.4	0.06	0.45	0.976	0.58	1.7	0.4	0.917
UTPS/ISC_2_	4.6	0.07	0.26	0.9807	0.55	1.81	0.5	0.91
UTPS/ISC_2_/SICNCs	7.6	0.1	0.2	0.9881	0.56	1.79	0.9	0.9076

	Dubinin–Radushkevich isotherm model			Temkin isotherm model		
	q_DR_ (mg/g)	K_DR_ (mol^2^/J^2^)	R^2^	b_T_ (kJ/mol)	K_T_ (L/mg)	R^2^
UTPS	3	4 × 10–6	0.8759	2.43	0.556	0.9564
UTPS/ISC_2_	3.16	3 × 10–6	0.8788	2.34	0.68	0.9598
UTPS/ISC_2_/SICNCs	5	1 × 10–6	0.8515	1.5	1.1	0.9629

### Mechanical characteristics

**Fig. 10:**
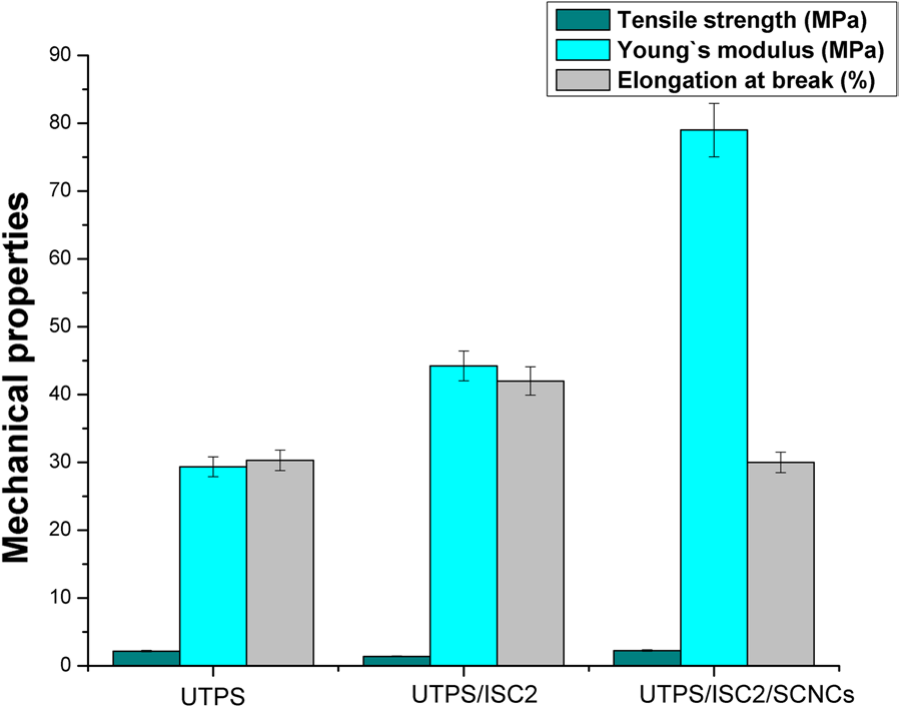
Comparable statistical analysis for mechanical characteristics of prepared starch-based composite films constructed using data obtained from Table 5. Bars represent the standard error of mean (n = 3)

The dispersion and compatibility of one component with another are critical, as these factors have a substantial impact on evaluating the mechanical features of composite materials. A significant advancement in the mechanical characteristics can be achieved by establishing a homogeneous dispersion of the reinforcing filler in the matrix associated with good interfacial adhesion [60, 61]. The mechanical properties of fabricated starch-based composite films characterized by the tensile tests were examined and shown in Fig. 10 and Table 5.

**Table 5:** Mechanical properties of as-synthesized starch-based films: Yield strength (Y), tensile strength (σ_max_), Young’s modulus (E), elongation at break (ε) and stress (σ) at break

Specimen	Strength (MPa)		E (MPa)	ε at break (%)	σ at break (MPa)
	Y	σ_max_			
UTPS	1.2	2.2	29.3	30.3	1.8
UTPS/ISC_2_	0.84	1.4	44.2	42	0.6
UTPS/ISC_2_/SICNCs	0.83	2.3	79	30	1

It is known that a high value of Young’s modulus (E) indicates low elasticity and high hardness properties [62]. According to Table 5, the addition of urea as a plasticizer significantly affected the elastic modulus of UTPS. The presence of urea reduced elasticity and formed TPS-based films stiffer and more resistant than TPS plasticized with polyol plasticizers (glycerol) [63]. Information on the impact of ISC_2_ and SICNCs merged into starch films is shown in Fig. 10. There was a substantial increase in the Young’s modulus from 29.3 MPa to 44.2 and 79 MPa, respectively, for the films of UTPS, UTPS/ISC_2_, and UTPS/ISC_2_/SICNCs. This modification caused an increase in the hardness of the starch-based films produced. The tensile strength decreased by 35% with UTPS/ISC_2_, but it slightly increased with UTPS/ISC_2_/SICNCs, with a ratio of 4.2% comparable to UTPS, and the most rigid structure was obtained with UTPS/ISC_2_/SICNCs. Since the starch acetylation reaction advocates incomplete hydrolysis, it also breaks the glycosidic bond on the starch molecular chains. As a result, the molecular weight of starch reduced resulting in a shorter molecular chain, thus the UTPS/ISC_2_ tensile strength decreased. On the contrary, the elongation at break increased dramatically from 30.3 to 42% for UTPS and UTPS/ISC_2_, adequately and remained constant for UTPS/ISC_2_/SICNCs, as seen from Table 5 and Fig. 10.

The acetyl group in UTPS/ISC_2_/SICNCs acted as a spacer and prevented the starch chains from getting too close, resulting in more interstitial space between the starch molecules [64]. Compared with the UTPS/ISC_2_ film, the SICNCs can capture this space and enhance the interaction with polymers, increasing the integrity of the film structure, as a consequence its tensile strength raised. Moreover, the enhancing effect of SICNCs on UTPS could be related to good dispersion and strong compatibility between UTPS and SICNCs in the film matrix due to the presence of hydroxyl groups and the formation of hydrogen bonds. Additionally, the nanosized cellulose allowed good surface contact with the starch matrix. Nevertheless, the chemical similarities between starch and cellulose can lead to a significant intermolecular hydrogen bonding interaction between the molecules [65]. In response, the SICNCs adherence to the film matrix was intensified [62], which resulted in increasing the Young’s modulus and tensile strength of the obtained UTPS/ISC_2_/SICNCs compared to other starch-based films.

## Conclusion

Corn starch was modified via the esterification process with iodine as an oxidizing agent to fabricate iodinated starch citrate (ISC). Thermoplastic starch (TPS) was manufactured using urea as a plasticizer applying the casting solution process to produce the following samples, UTPS, UTPS/ISC_2_ and UTPS/ISC_2_/SICNCs. The inclusion of ISC_2_ and SICNCs into TPS films improved the water stability of biopolymer starch. The higher water vapor absorption rate in UTPS and UTPS/ISC_2_, the greater the controlled barrier property. UTPS/ISC_2_/SICNCs film demonstrated insoluble characteristics and no water solubitity at room temperature in spite of its poor water vapor absorption profile. Furthermore, the reduction in S_BET_ measurements associated with the exfoliation of ISC_2_ and SICNCs within the polymer matrix illustrated their ability to improve the plasticization of starch-based films, as evidenced by SEM micrographs revealing an extensive destruction of the morphology of the produced starch granules. The addition of ISC_2_ and SICNCs fillers to UTPS enhanced the Young’s modulus from 29.3 to 44.2 and 79 MPa for UTPS/ISC_2_ and UTPS/ISC_2_/SICNCs respectively, resulting in a higher hardness. The analysis of the adsorption isotherms showed that the R^2^ values matched the following order: Langmuir > Temkin > Freundlich > Dubinin-Radushkevich. The UTPS/ISC_2_/SICNCs showed the highest removal (%) of 83% for CV dye uptake.

## Data Availability

All data generated or analyzed during this study are included in this published article.
